# Pathological Distal Tibial and Fibular Fracture in a Paediatric Patient: A Case Report

**DOI:** 10.7759/cureus.30235

**Published:** 2022-10-12

**Authors:** Muhammad Mubashir Siddiqui, Anand Pillai

**Affiliations:** 1 Department of Trauma and Orthopaedics, Wythenshawe Hospital, Manchester, GBR

**Keywords:** non-ossifying fibroma, open reduction internal fixation, distal fibula, distal tibia fracture, synthetic bone graft, bone lesion, bone grafts, orthopaedics trauma, paediatrics orthopaedics, orthopaedics surgery

## Abstract

Paediatric distal tibial and fibular fractures are seen quite regularly in orthopaedic trauma practice. Most patients are managed conservatively with closed reduction or casting while only a selected few required surgical treatment. Surgical options include plating, percutaneous Kirschner wires, rigid intramedullary nails, and flexible intramedullary nailing. This is dependent upon the patient’s age, fracture site, comminution, and concomitant injuries. Here, we present an interesting case of a patient with an unusual lesion seen at the fracture site. This lesion was curetted out during surgery and filled with an injectable synthetic Cerament bone void filler (Bone Support AB, Lund, Sweden), which later formed into bone and allowed the bone to remodel.

## Introduction

Paediatric tibial fractures represent 15% of all paediatric fractures and are the third most common paediatric long bone fractures after the femur and radius-ulna [1]. Of these, approximately 30% are associated with a fibular fracture [2]. Management of these fractures is dependent on several factors. However, most of them are managed conservatively with closed reduction and casting, while only a select few require surgical treatment. This case report presents a patient with an unusual lesion seen at the site of the distal tibial fracture site. Traditionally, autologous bone grafts are used to fill bone voids. This case report shows the use of a novel injectable synthetic Cerament bone void filler (Bonesupport AB, Lund, Sweden) used in the treatment of such lesions. The patient was regularly followed up in the clinic post-operatively where he had a specialised regimen for foot support and weight-bearing. Serial scans done in the follow-up period show gradual healing of the fracture. X-rays done 18 months post-operatively showed full healing of the fracture and complete consolidation of the lesion.

## Case presentation

**Figure 1:**
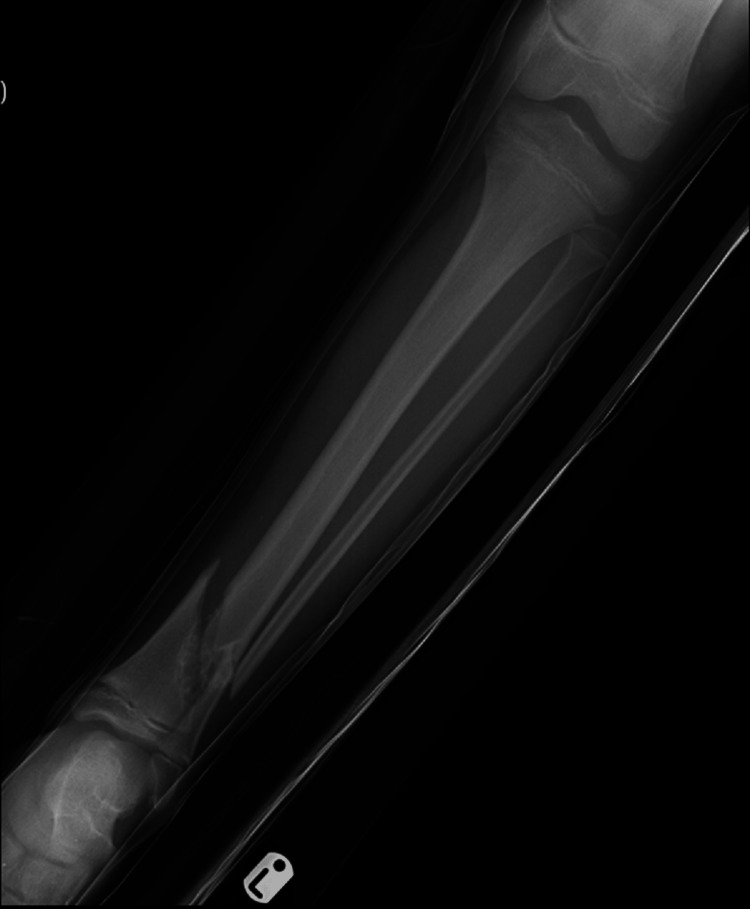
Anteroposterior (AP) view of the left tibia and fibula

**Figure 2:**
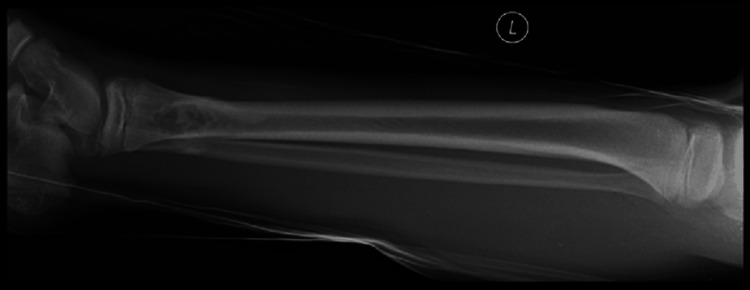
Lateral view of the left tibia and fibula

A 10-year-old boy presented to the emergency department having sustained a fracture in the summer of 2016. He had a background of asthma and an allergy to nuts. His only previous surgery was a left ear foreign body removal. He was skipping on and off the pavement and injured his left ankle. The injury was described as a twisting inversion injury by the patient and his mother. On initial examination, he was complaining of pain and swelling in the left ankle which had an obvious deformity and associated bruising. It was a closed and isolated injury with an intact distal neurovascular status. An initial X-ray of the left ankle showed an oblique fracture of the distal tibia and fibula. However, interestingly the distal tibia demonstrated an underlying bubbly cortically based lesion in keeping with a benign non-ossifying fibroma (Figures 1, 2).

**Figure 3:**
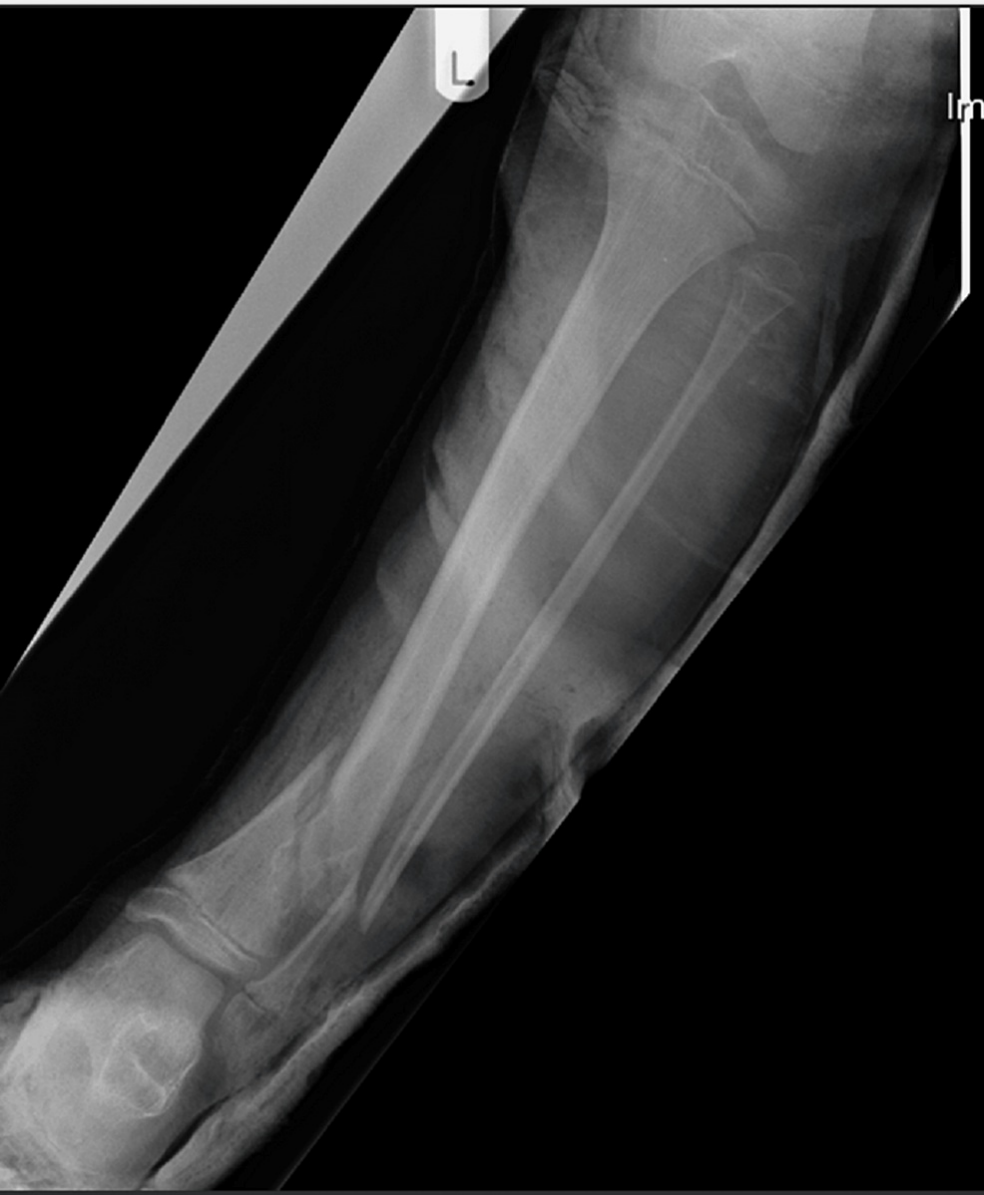
AP view of the left tibia and fibula post-backslab AP: anteroposterior

**Figure 4:**
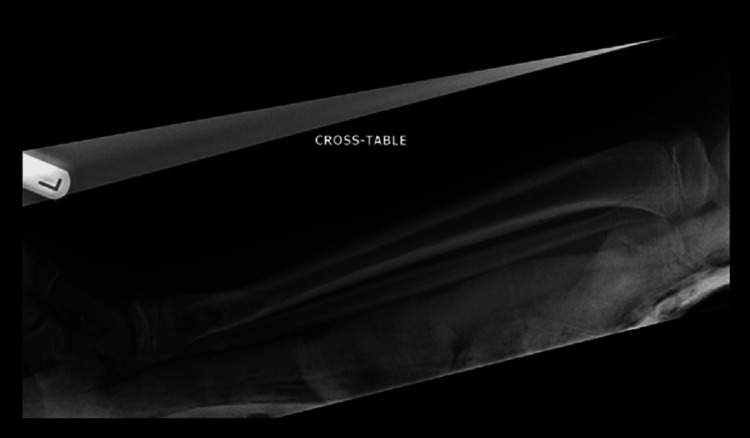
Lateral view of the left tibia and fibula post-backslab

Initial management involved pain control with basic analgesics and application of a backslab. Repeat X-rays taken in the backslab showed a pathological fracture through the distal tibial metaphysis with an associated fibular fracture (Figures 3, 4). The underlying lesion was eccentric, well defined with sclerotic margins and a narrow zone of transition. The appearances were in keeping with a benign lesion. The differential diagnoses included a non-ossifying fibroma or an aneurysmal bone cyst.

**Figure 5:**
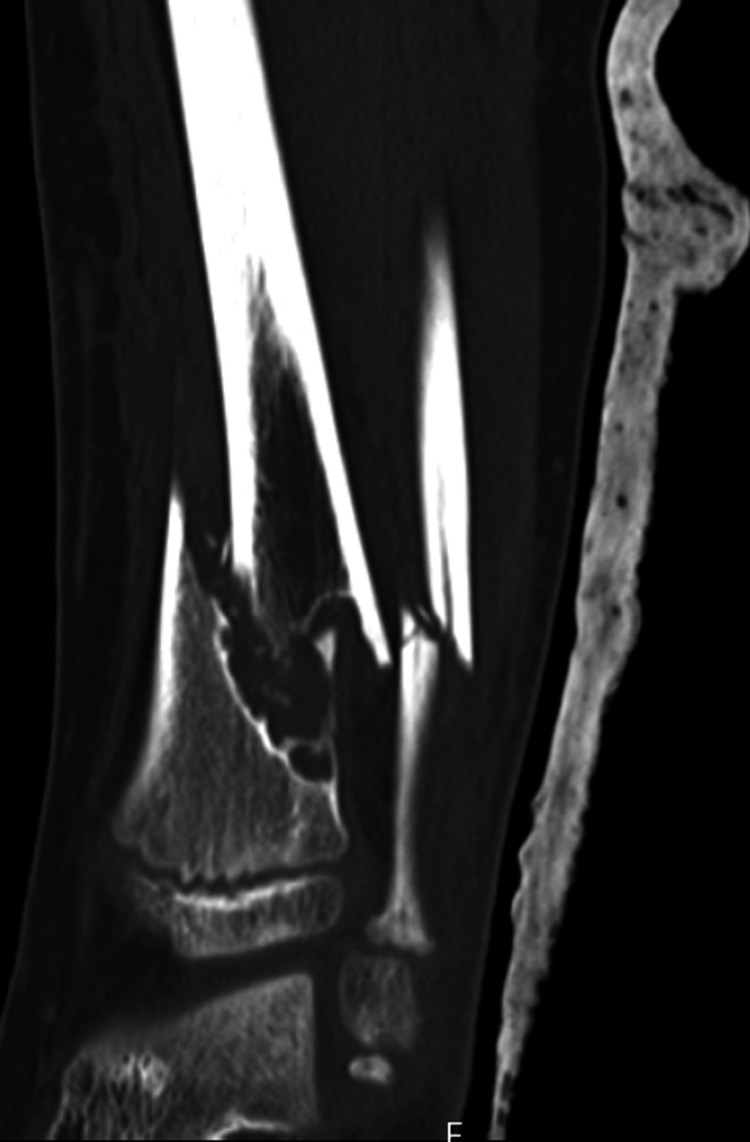
Coronal CT view of the left tibia and fibula

He later underwent a computed tomography (CT) scan which confirmed the previous findings of a pathological closed oblique fracture through a primary bone lesion involving the distal one-third diaphysis of the tibia with an associated oblique fracture of the fibula (Figure 5). The distal fracture fragments were anteriorly displaced with overlying soft tissue swelling. The joints above and below were intact. The primary bone lesion was eccentric, and juxta cortically based, lucent with a bubbly lobular outline most in keeping with a non-ossifying fibroma. In conclusion, it demonstrated a pathological distal third tibia and fibula fracture through a non-ossifying fibroma.

**Figure 6:**
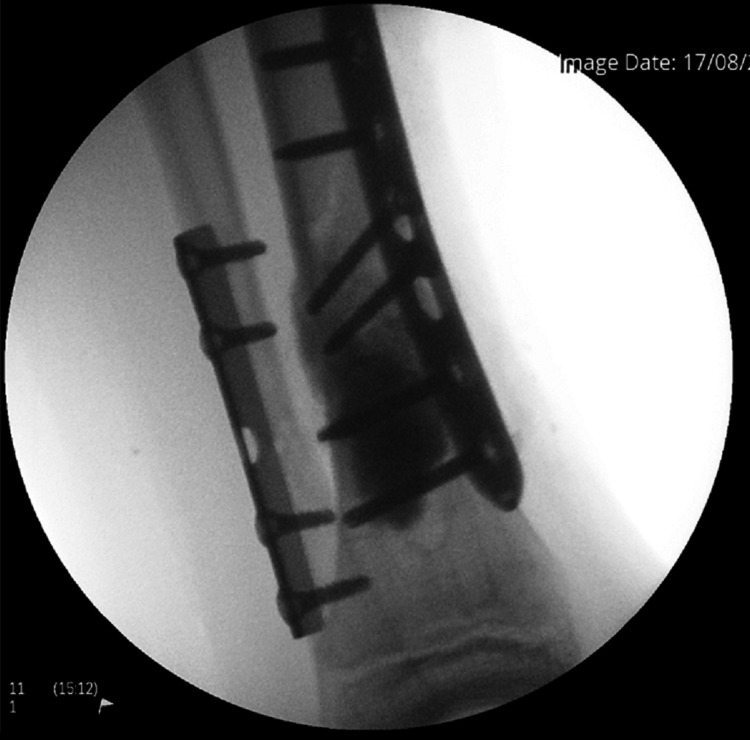
Fluoroscopy image from left tibia and fibula ORIF ORIF: open reduction internal fixation

The patient later underwent an open reduction internal fixation (ORIF) of his distal tibia and fibula. Plate osteosynthesis was carried out using a distal tibial and fibular plate with synthetic bone grafting. This was a 100-degree tubular fibular five-hole plate with four cortical screws. The lesion was curetted out and a 5 cc (5 ml) Cerament bone void filler was used to fill the void. This was then fixed with a 3.5 mm compression locking six-hole plate where four cortical locking screws were used. Two screws were placed in the tibia. A good and acceptable reduction was achieved, and check X-rays were performed (Figure 6). The wound was closed with polyglactin and poliglecaprone. The growth plate was protected throughout the procedure. Samples from surgery sent for histology showed some necrotic tissue, small fragments of bone, granulation tissue and blood clots, but no evidence of a malignancy.

**Figure 7:**
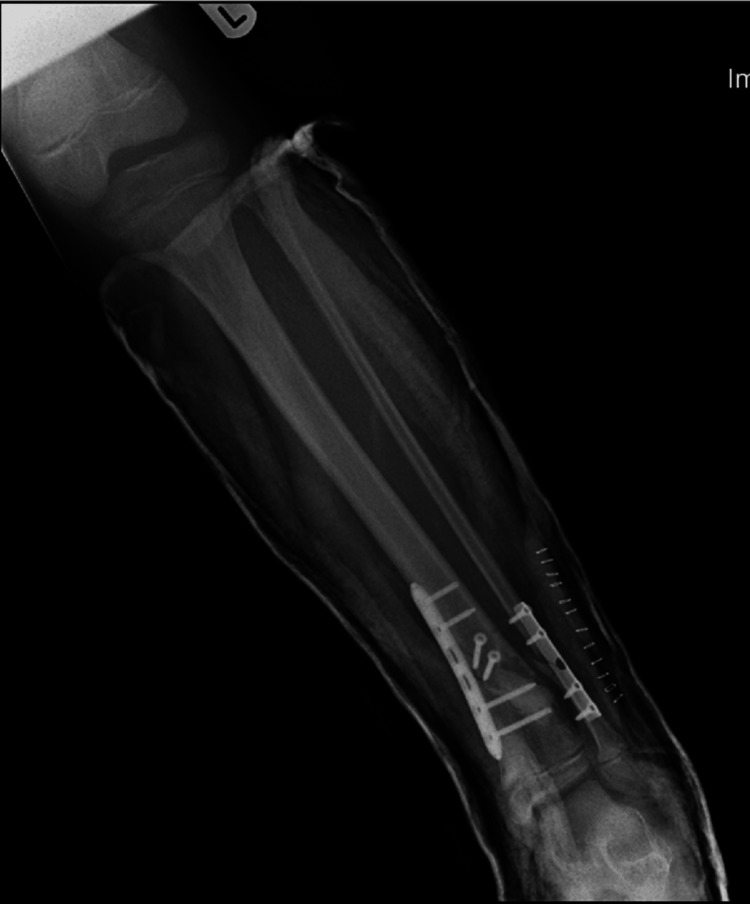
AP view of the left tibia and fibula post-surgery in a below-knee cast AP: anteroposterior

**Figure 8:**
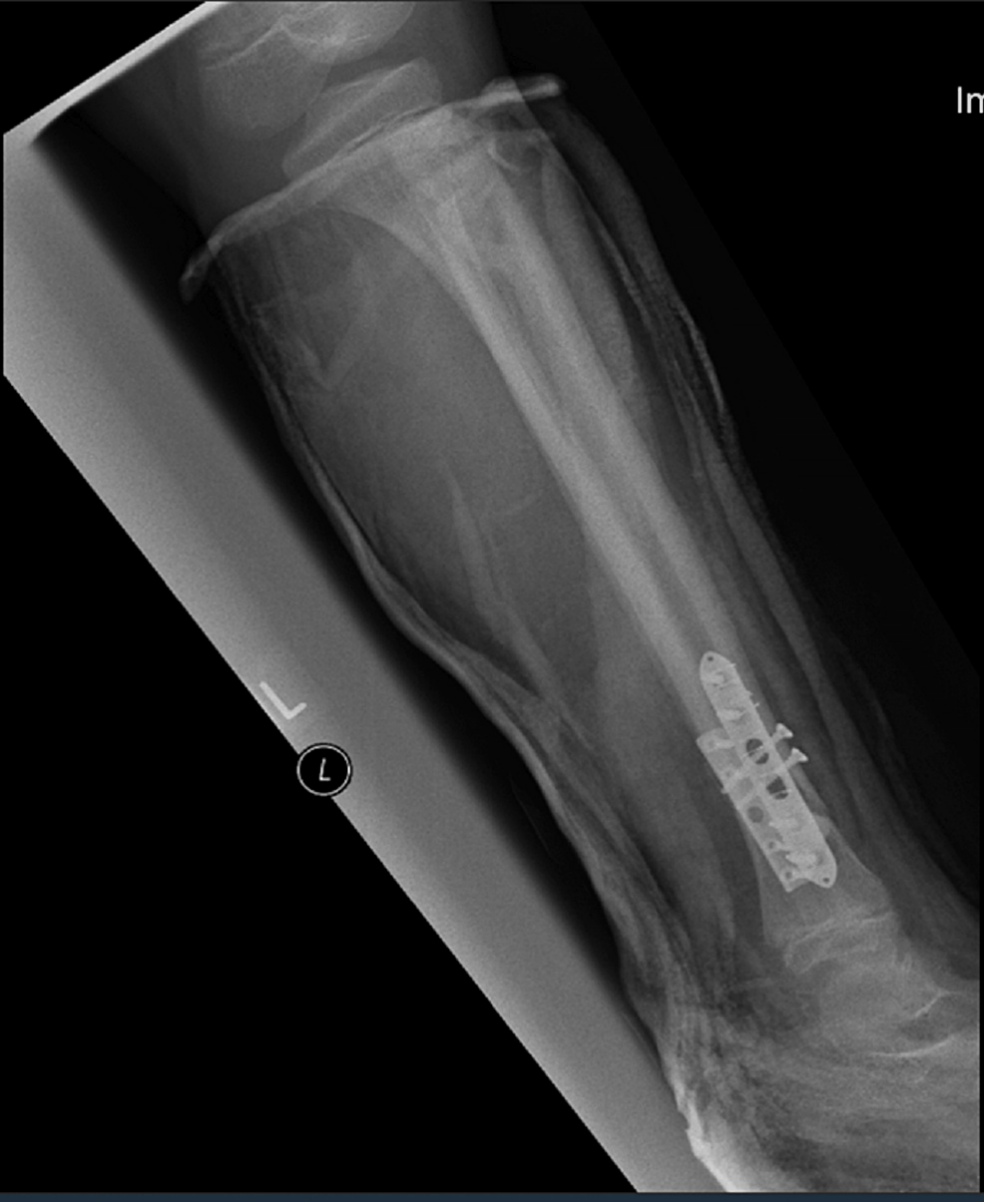
Lateral view of the left tibia and fibula post-surgery in a below-knee cast

A full below-knee cast was applied post-surgery (Figures 7, 8).

He was later discharged from the hospital following surgery and was followed up in the clinic. He was managed in a below knee non-weight bearing cast for nine weeks. At this point, he was placed in a below-knee lightweight cast and allowed to partially weight bear. He was then moved to a moon boot and allowed to partially weight bear through this at 14 weeks post-surgery.

**Figure 9:**
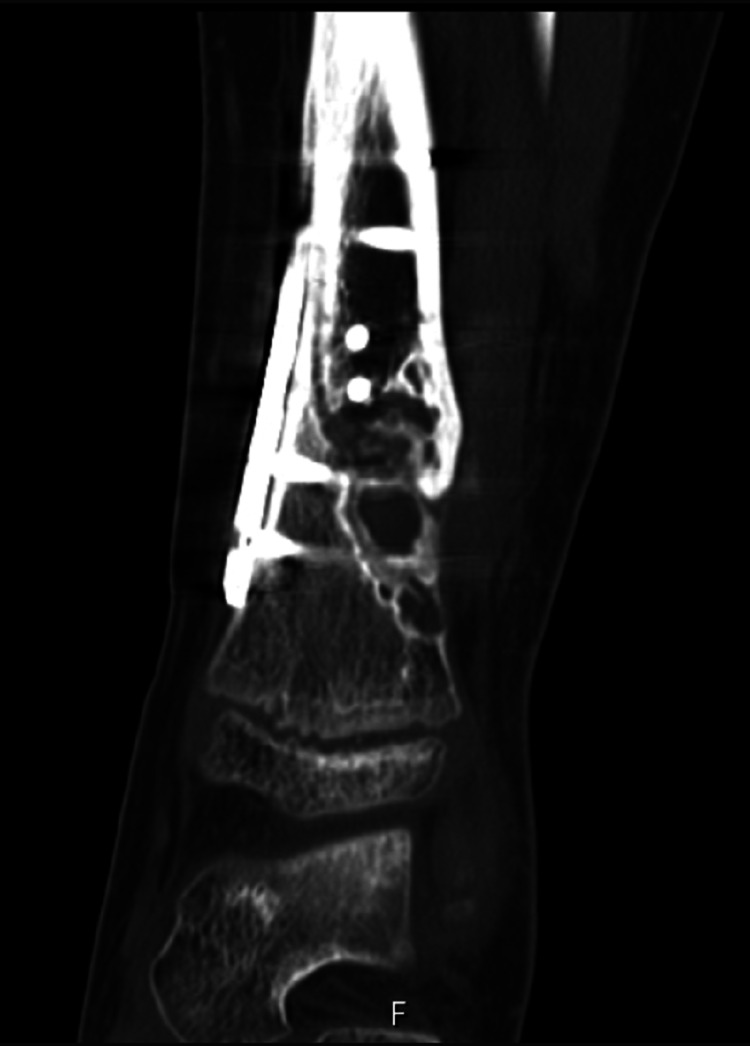
Coronal CT view of the left tibia and fibula

A CT scan performed 18 weeks post-op showed only 60% of bone healing (Figure 9). This was done to comprehensively assess the ability of the synthetic Cerament bone void filler to form new bone and remodel into bone. We can already see from this CT scan done four-and-a-half months after the surgery that the Cerament had started remodelling into bone.

**Figure 10:**
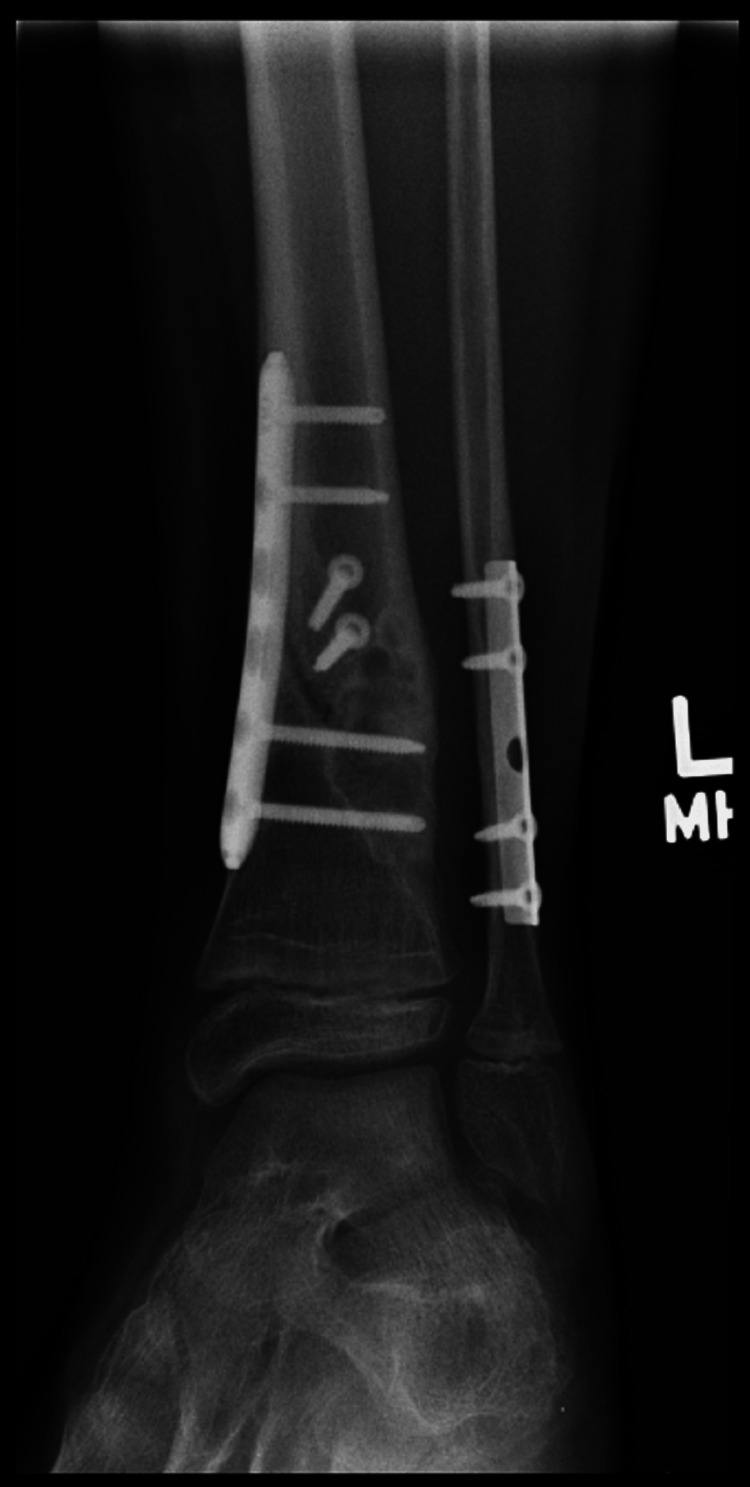
AP view of the left tibia and fibula AP: anteroposterior

**Figure 11:**
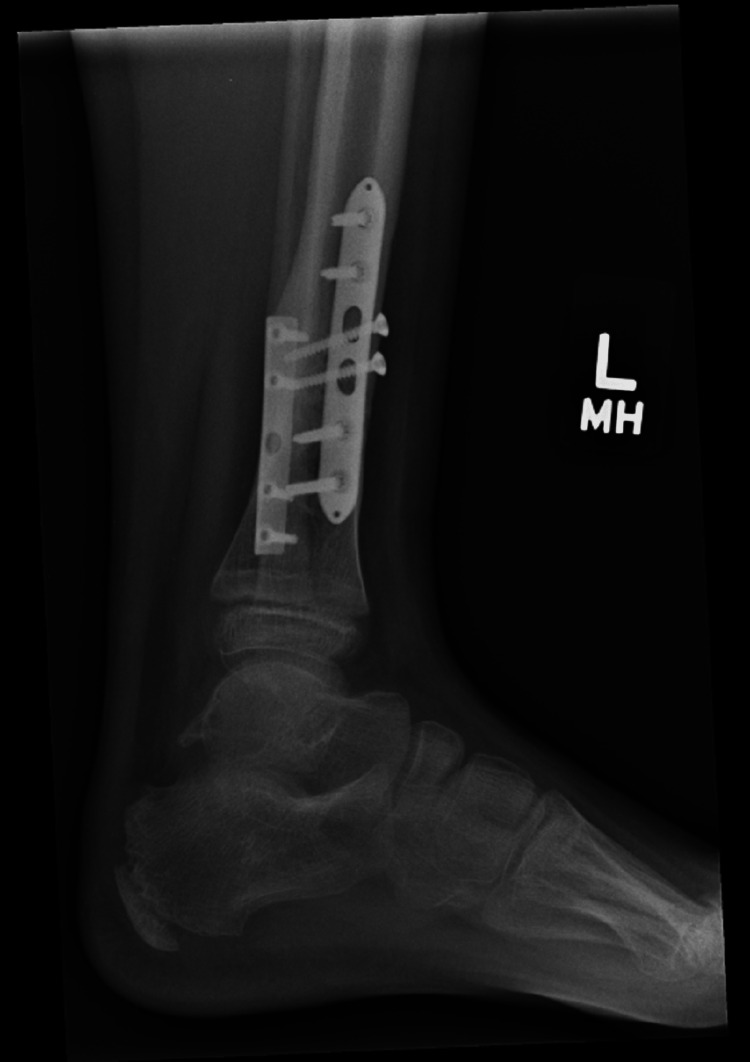
Lateral view of the left tibia and fibula

At 24 weeks post-op, he was fully weight-bearing with no pain and had a full range of movement in the left ankle. X-rays done at that point showed further progression of healing (Figures 10, 11). He was then allowed to fully weight bear and only advised to wear the moon boot outdoors.

**Figure 12:**
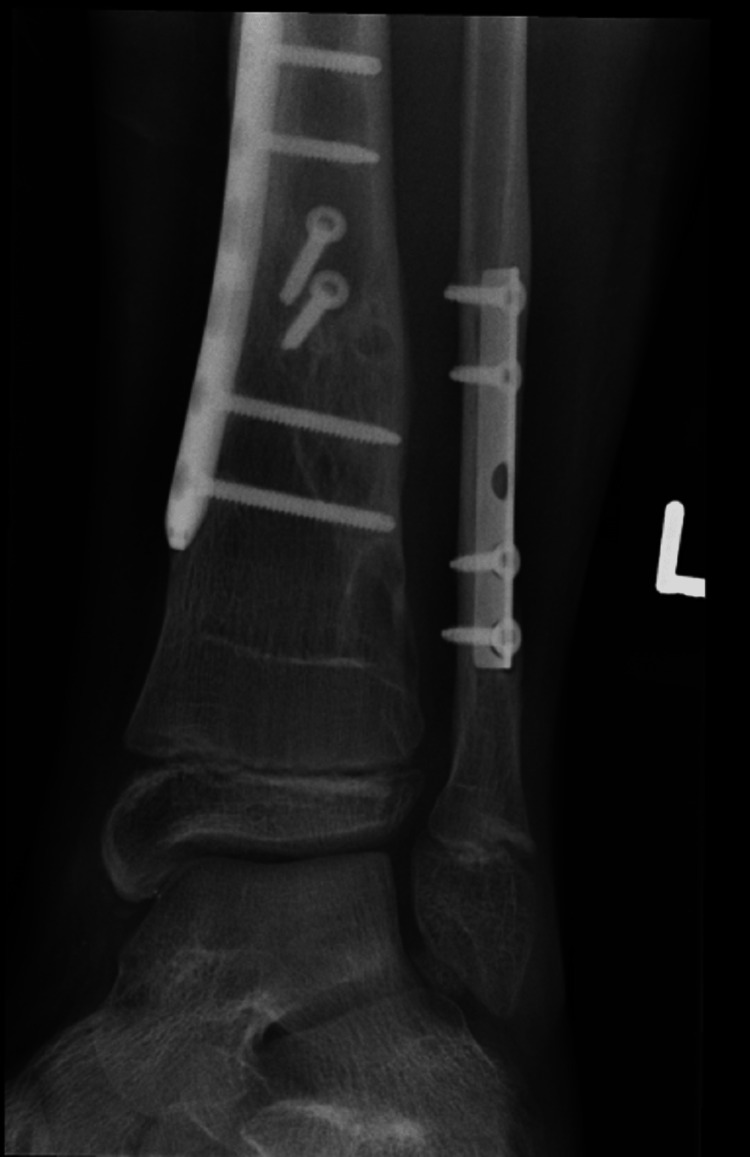
AP view of the left tibia and fibula AP: anteroposterior

**Figure 13:**
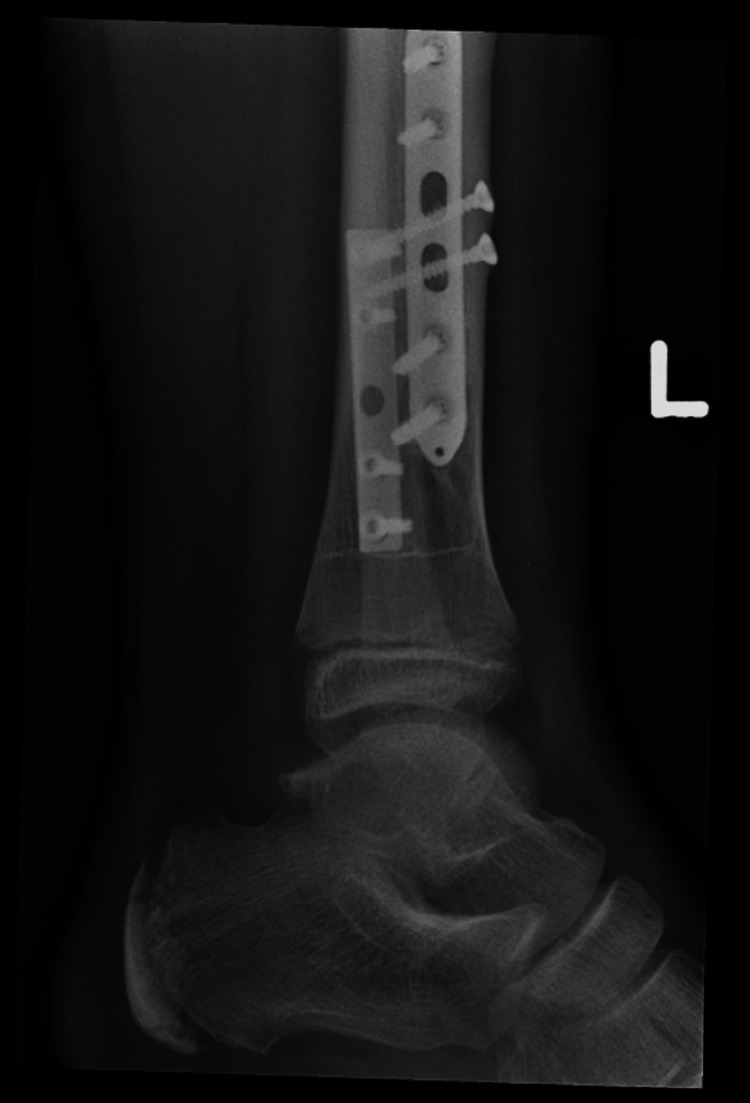
Lateral view of the left tibia and fibula

Further X-rays done upon arrival in the clinic 18 months post-op showed full consolidation of the cyst. The fracture had fully healed with no issues at the wound site (Figure 12, 13).

**Figure 14:**
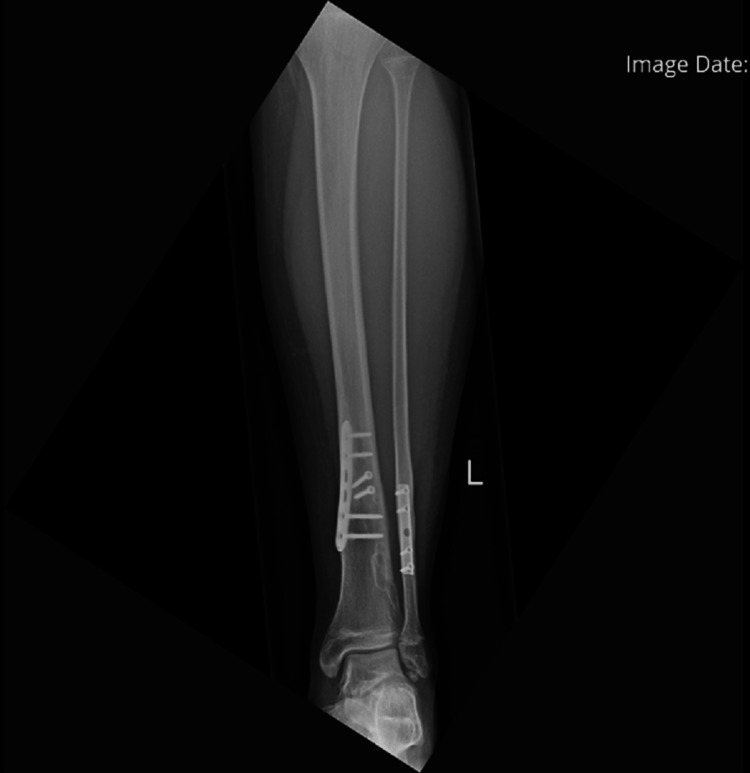
AP view of the left tibia and fibula AP: anteroposterior

**Figure 15:**
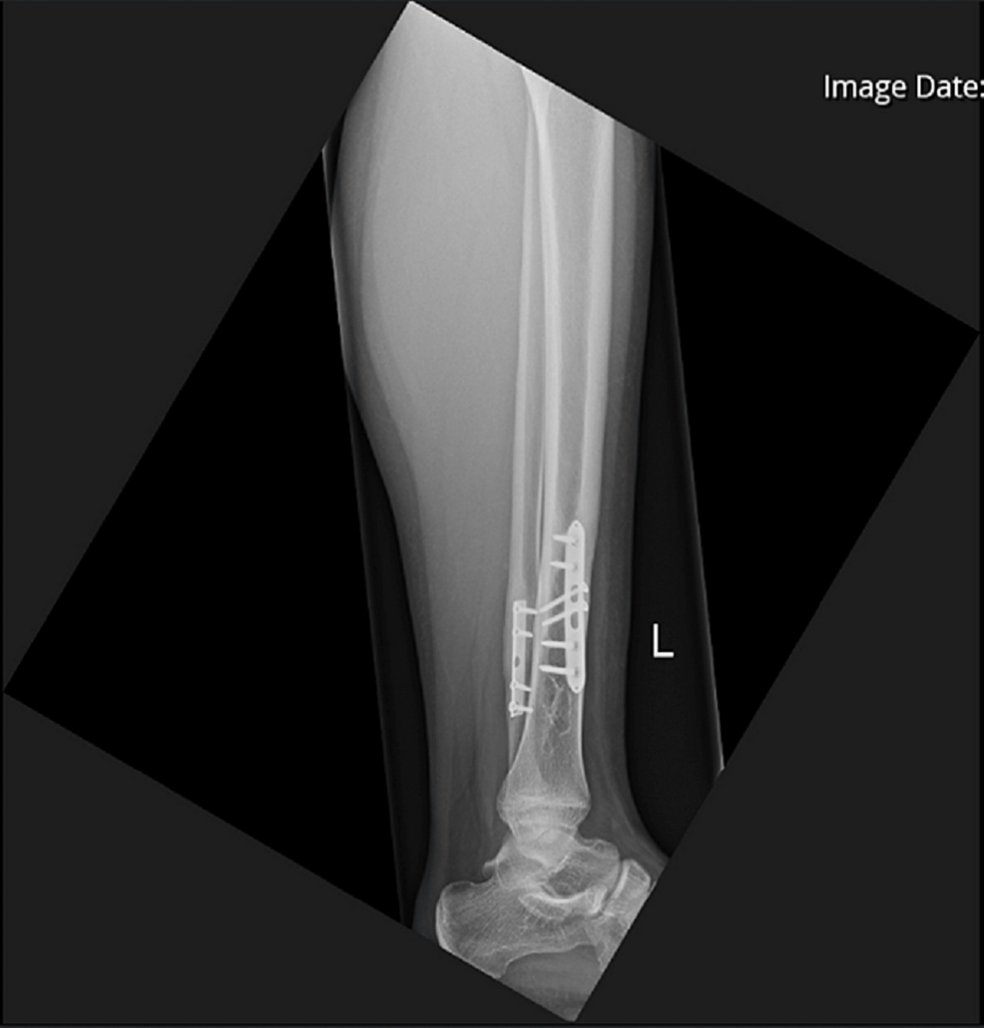
Lateral view of the left tibia and fibula

He was referred to the clinic six years after his initial injury for consideration of plate removal. He complained of occasional swelling and pain around his previous fixation site but denied daily pain. X-rays done at this point showed that the plates had been incorporated by the bones (Figures 14, 15). Due to the relative risk and benefit ratio, the decision was made to not remove the plate.

## Discussion

Ossifying fibroma of the long bones, or osteofibrous dysplasia (OFD), is a fibro-osseous developmental bone condition that commonly occurs in the tibial mid-shaft in children [3,4]. It is usually found in children under the age of 10, with males affected more than females [5]. It is mostly asymptomatic but can present with a painless swelling over the anterior tibia. Rarely it can present with a pathological fracture and can be picked incidentally on X-rays done due to other indications [5]. Typical radiographic findings of OFD include well-circumscribed, eccentric, osteolytic lesions with a sclerotic border in the anterior tibial diaphysis [6].

OFD is a rare disease with limited published literature. There are no definitive management recommendations and treatment options can be controversial. Some surgeons recommend a biopsy only followed by observation and conservative management. Surgical treatment is indicated in pathological fractures or deforming lesions [6]. Surgical options include curettage or localised subperiosteal excision, but both are related with a risk of recurrence [7].

When curettage is conducted, the bone cavity needs to be filled with acrylic cement or bone graft [8]. In the case presented above, Cerament bone void filler was used, which is a mouldable, injectable, and drillable synthetic bone void filler consisting of 60% calcium sulphate, 40% hydroxyapatite and a radio-contrast agent called iohexol. Calcium sulphate acts as a resorbable carrier for hydroxyapatite, which is highly osteoconductive and promotes bone ingrowth. This bone void filler remodels into host bone within 6-12 months [9].

We can see from the serial X-rays and post-op CT that new bone continued to form. At 18 weeks post-op, the CT scan showed 60% bone healing. The patient was already fully weight-bearing at 24 weeks post-op. This demonstrates the ability of the Cerament bone void filler to form new bone and remodel into bone.

## Conclusions

Pathological fractures can often be the initial presentation of a bone lesion. Bone lesions are often asymptomatic and picked up incidentally on X-rays. The presence of a bone lesion can change the management of what otherwise would have been an uncomplicated fracture. Traditionally, autologous bone grafts were used to fill this void. This case report is evidence that a calcium sulphate and hydroxy apatite synthetic bone graft, used as a substitute to autologous bone grafts, produce favourable results and have the ability to form bone and remodel into bone.
